# The Victory of King Edward's Hospital Fund for London

**Published:** 1919-07-12

**Authors:** 


### THE VICTORY OF KING EDWARD'S HOSPITAL FUND FOR LONDON.

The Egyptian Hall of the Mansion House has been the scene of many memorable banquets, but it is the fact that amongst them all the banquet given on Thursday, July 3, 1919, by Colonel the Right Hon. Sir Horace Brooks Marshall, LL.D., Lord Mayor, to His Royal Highness the Prince of Wales, K.G., (the President), the Council, the Committees, and the Visitors of King Edward's Hospital Fund for London, may surpass them all in permanent value and importance to the people of the Metropolis of the Empire to-day. The Mansion House since it was erected between 1739 and 1753 has been increasingly the home of philanthropy of the best within the British Isles. Many important movements of great public utility have originated within its walls, and since 1873, when the churches of all denominations in London commenced to support collectively on one Sunday in the year the voluntary hospitals, thus following the example of Birmingham where the late Canon Miller's initial Hospital Sunday was first put into practice in 1859, the Mansion House has been a continuous supporter, upholder and friend, through successive Lord Mayors, of the Metropolitan Hospital Sunday Fund. In 1873, its first year, the Sunday Fund realised £27,700 for the London hospitals, which increased in 1918 to £92,055, and it is hoped it will yield at least £100,000 in 1919. In the forty-six years during which the churches through this Fund have worked in support of the voluntary hospitals the latter have received through its agency, including 1918 collections, no less a sum than £2,331,665.

But the devotion and generalship of those responsible for the maintenance and working of the Hospital Sunday Fund are aptly illustrated by an incident which happened in the year 1875, when the surgeon of a relatively small special hospital set himself to overthrow the Fund, and filled the Egyptian Hall of the Mansion House with the past and present out-patients at his institution, with the object of changing the Fund's policy at the risk of destroying it. In the result he succeeded in carrying by a large majority a resolution hostile to the Council and its Managers, and was so intoxicated by his success that the present writer saw him, upheld by two of his supporters, literally reeling as he made his way through the saloon of the Mansion House into the street. This incident was rendered possible by the fact that the Sunday Fund in those days had no constitution, and that when a meeting was held every man in the street, as he passed the Mansion House, could enter the Egyptian Hall in attendance at a meeting of the Fund, and vote as he pleased. The consequences of the packed meeting referred to were disposed of by the Council and supporters of the Sunday Fund agreeing to a Constitution, holding a meeting under it, and so putting the Fund and its proceedings on a sound and constitutional basis.

Continuous efforts were made for many years afterwards to raise the Sunday Fund to £100,000 a year, but that total was never approached until in 1907 the late George Herring left a large sum to be distributed through the Sunday Fund, which brought the total receipts in that year to £78,651. Till this date the receipts had only once exceeded £64,000, and the voluntary hospitals of London needed £100,000 more than this from voluntary gifts in 1897, the year of the Diamond Jubilee of Queen Victoria. If the voluntary system was to continue and thrive at least this sum was urgently needed, as the supporters of the system fully realised. Her Majesty declined to express a preference for any one of the many schemes (some 4,000) set on foot or suggested as expressions of loyalty.

Her Majesty thus left the Prince of Wales at liberty to press upon the inhabitants of London a project in which he took a keen interest. Under this scheme King Edward, of blessed memory, hoped to turn into a channel of permanent beneficence the sentiments called forth by the contemplation of a reign of sixty years fraught with blessings. In associating his name with this effort, the late King Edward displayed a commendable courage, foresight, and judgment. To demonstrate that this was so we may first point out that for some years, culminating with the supreme effort made by The Hospital in 1895, when £60,361 was raised through the Sunday Fund, continuous efforts to obtain for the hospitals every year at least £100,000 had been made. The total receipts for 1896, confirmed and seriously emphasised as they subsequently were by those of 1897 and 1898, demonstrated how serious the financial outlook of the voluntary hospitals of London in reality was. This depletion was, however, checked, and the financial position of the Sunday Fund's collections was improved by the institution of King Edward's Hospital Fund, for in 1899 its total receipts increased to £53,504, and ever since they have continued to grow on an average every year, until they now approximate, if they do not exceed, £100,000 a year.

Our study of the inertness in the giving public of London to its hospitals lead us to conclude that it was largely due to the want of a crusade—a sort of Peter the Hermit—to teach and preach the privilege of personal service in the days of health in the cause of the sick. Few, indeed, at this time realised the power resting in such personal service, a fact which at that time had been missed by the churches, and is to-day far too little recognised and encouraged. One great organ of public opinion, when it was mooted abroad that there was a possibility of a great scheme of collecting money for the benefit of the London hospitals on the part of the Prince of Wales, in celebration of the Queen's reign, hastened to warn his Royal Highness of the *risks of disaster which lay before him.* "About the importance of the object in view there could

**The Victory of King Edward's Hospital Fund for London**—(*continued*).

not be any difference of opinion, *but in taking it into practical consideration, its magnitude and the conditions of the time at which it must be launched were factors which must not be lost sight of.*" In the year 1896 his Royal Highness had set Guy's Hospital upon its feet by a supreme effort and splendid organisation, which was crowned by a dinner at the Imperial Institute, which produced annual subscriptions and gifts amounting to the splendid equivalent in capital of some £360,000, the greatest result from a dinner which the world has ever seen. The Press critic, however, speaking generally, wrote: "It may be said that a second effort such as that on behalf of Guy's, whilst it might be expected to call forth a favourable response" if it was confined to the current year, was a much more serious matter if what was in contemplation was a permanent endowment of considerable magnitude, which would really set the London hospitals on their feet. This was a formidable enterprise for various reasons duly set forth. The conclusion, boldly published a few days before the King's Hospital Fund was launched, was "It would be exceedingly unfortunate if the Prince of Wales, in his laudable desire to promote the interests of such admirable institutions as the London hospitals, should be led into any undertaking of which the success is not reasonably assured. To make any sort of substantial permanent provision for the London hospitals would be a far larger financial operation than establishing the Imperial Institute." The time surrounded the effort with grave disadvantages, to wit, that of coming after the Imperial Institute effort, of having to compete with other general and local demands upon the public liberality, of coming in the same year as the Indian famine, and, last if not least, "of making an appeal for a scheme the details of which are not before the public, although the excellence of its ultimate object is universally admitted."

Such, then, was the position of affairs on February 3, 1897, thirteen days after a meeting of representatives of men of the day and of all types of public opinion, which the Prince of Wales had summoned to Marlborough House to consider the scheme. The late King Edward was fully alive to the special difficulties and opposing forces with which the scheme was threatened, but before entering the room of assembly he expressed with purposeful earnestness how near the proposal was to his heart, and assured the men he had consulted of his confidence in their judgment and efforts, and reliance upon their gaining the support of this meeting.

### OUR DEBT TO KING EDWARD.

The nation and the people of these Islands are thus placed in possession of facts, which demonstrate in a remarkable way the debt which the hospitals of London, and indirectly the voluntary hospitals throughout the country, owe to the late King Edward. The King's Hospital Fund attained its majority in 1918, and at December 31 in that year the total sum received and paid into the bank of the Fund was £5,136,586, of which £2,666,576 had been awarded and paid to hospitals, convalescent homes, and consumption sanatoria. The total invested funds of the King's Hospital Fund at December 31, 1918, amounted to £2,505,615. Could the heartfelt, purposeful, courageous efforts of a Great and Beloved King in the welfare and interests of his poorer subjects, especially, have greater or more complete justification than King Edward's insistent action in connection with Queen Victoria's London Jubilee Memorial, as the facts here given convincingly demonstrate? It is interesting to add that the total expenses for the twenty-one and three-quarter year's working are only £63,865, being an average of £2,903 per annum.

### THE LORD MAYOR'S BANQUET IN THE EGYPTIAN HALL, JULY 3, 1919.

His late Royal Highness's aim in establishing King Edward's Hospital Fund for London in 1897, was not only to secure by voluntary contributions a large annual revenue for the maintenance of the voluntary hospitals of the Metropolis of the Empire, but to give this Fund an active vitality, resting on the heartfelt sympathies and spontaneous generosity of its inhabitants, combined with a council and committees, the principles of which would represent fully and impartially all sections of the people. It was not possible in the circumstances of the moment to secure the presence at the Banquet on July 3 of everyone who was connected with the management of this great Fund. One notable and important absentee was the Speaker of the House of Commons, the Right Hon. James W. Lowther, M.P., who has for nine years rendered valuable service as one of the Governors of the Fund during the Prince of Wales's minority, and many times Chairman of the General Council Meetings. His co-governors, the Marquess of Cambridge and Viscount Iveagh, the Marquis of Crewe (Lord-Lieutenant of the County of London), Lord Revelstoke, treasurer and chairman of the finance committee, the Hon. N. Charles Rothschild, and a few other members of the general council were unavoidably prevented from being present. The following list of those present will clearly indicate, when it is pointed out that with some five exceptions all the original council of the Fund have joined the majority, how the representative character of the council, the committees, and visitors has been maintained.

### NAMES OF THE GUESTS.

There were present H.R.H. The Prince of Wales, K.G., Col. The Rt. Hon. The Lord Mayor, LL.D.; The Lady Mayoress, Miss Marshall, Captain Hon. Piers Legh, and the following

### MEMBERS OF GENERAL COUNCIL AND/OR COMMITTEES:

The Bishop of London, K.C.V.O., His Eminence Cardinal Bourne; The Very Rev. the Chief Rabbi; The Earl of Bessborough, K.P.; The Viscount Knutsford; The Viscount Mersey; The Viscount Farquhar; Col. Viscount Burnham, C.H.; Lieut.-Col. Lord Stamfordham, G.C.B., G.C.I.E., G.C.V.O.; Lord Murray of Elibank; Lord Somer-

**The Victory of King Edward's Hospital Fund for London**—(*continued*).

leyton, K.C.V.O.; Lord Stuart of Wortley, K.C.; The Rt. Hon. Christopher Addison, M.P., M.D. (Minister of Health); Lord Downham (Chairman of the County Council); Sir Norman Moore, Bt., M.D. (President of the Royal College of Physicians); Major-General Sir George Makins, G.C.M.G. (President of the Royal College of Surgeons); The Rt. Hon. Sir T. Vezey Strong, K.C.V.O., K.B.E.; Sir William S. Church, Bt., K.C.B., M.D., F.R.C.P.; Sir Francis H. Champneys, Bt., M.D.; Lieut.-General Sir Alfred Keogh, G.C.B., C.H., F.R.C.S.; Sir Owen Phillips, G.C.M.G., M.P.; Sir Henry Burdett, K.C.B., K.C.V.O.; Sir William H. Bennett, K.C.V.O., F.R.C.S.; Sir William J. Collins K.C.V.O., M.D.; Sir Cooper Perry, M.D., F.R.C.P. (Vice-Chancellor of the University of London); Sir John Craggs, M.V.O.; Sir John Tweedy, F.R.C.S.; Sir Frederick M. Fry, K.C.V.O.; Mr. John G. Griffiths, C.V.O.; Mr. Yvon R. Eccles; Mr. Robert Fleming; Mr. George Lawson Johnston; Mr. W. J. H. Whittall; Sir Edward Hope, K.C.B.; Sir Walter Trower; Mr. Henry L. Hopkinson; Sir J. Rose Bradford, K.C.M.G., M.D.; Sir Frederick Green; Mr. Leonard L. Cohen; The Hon. Oswald Partington; Sir J. Kingston Fowler, K.C.V.O., M.D., F.R.C.P.; Dr. H. Morley Fletcher, F.R.C.P.

**HOSPITAL AND INSTITUTION VISITORS.**

*Medical.*—Miss L. B. Aldrich-Blake, M.D., M.S.; Lady Barrett, M.D., M.S.; Lt.-Col. W. H. Battle, F.R.C.S.; Mr. George Blacker, M.D., F.R.C.P.; Sir John F. H. Broadbent, Bt., M.D., F.R.C.P.; Mr. F. F. Burghard, C.B., F.R.C.S.; Mr. James Calvert, M.D., F.R.C.P.; Surg-Gen. Sir R. Havelock Charles, M.D.; Surg.-Gen. Sir G. Lenthal Cheatle, K.C.B.; Mr. Edred M. Corner, F.R.C.S.; Mr. Willmott Evans, F.R.C.S.; Mr. C. H. Fagge, F.R.C.S.; Mr. David Forsyth, M.D., F.R.C.P.; Dr. P. H. S. Hartley, C.V.O., F.R.C.P.; Mr. Henry Head, M.D., F.R.C.P.; Mr. G. Seccombe Hett, F.R.C.S.; Mr. Donald Hood, M.D., F.R.C.P.; Mr. Owen Lankester, M.R.C.S.; Mr. V. Warren Low, F.R.C.S.; Col. Mansell Moullin, M.D., F.R.C.S.; Mr. John Murray, F.R.C.S.; Col. T. H. Openshaw, C.B., C.M.G., F.R.C.S.; Lt.-Col. Sir D'Arcy Power, K.B.E., F.R.C.S.; Mr. Amand Routh, M.D., F.R.C.P.; Mr. H. Batty Shaw, M.D., F.R.C.P.; Mr. James Sherren, F.R.C.S.; Dr. Reginald V. Solly, F.R.C.S.; Mr. Herbert R. Spencer, M.D., F.R.C.P.; Mr. Walter G. Spencer, M.D., F.R.C.S.; Mr. F. J. Steward, F.R.C.S.; Mr. James H. Stowers, M.D.; Mr. Seymour Taylor, M.D., F.R.C.P.; Sir Nestor Rirard, M.D., F.R.C.P.; Surgeon Rear-Admiral Sir George Turner, K.B.E., C.B., F.R.C.S.; Col. H. J. Waring, F.R.C.S.; Mr. Guy E. Wood, M.B.; Mr. R. A. Young, M.D., F.R.C.P.

*Lay.*—Mr. H. C. Allen; Sir Godfrey Baring, Bt.; Major The Lord Blythswood; Mrs. Brodie; Major Sir Edward Coates, Bt., M.P.; Hon. George Colville; Mr. W. Ward Cook; Miss Winifred C. Cullis, D.Sc.; Mr. G. Acton Davis; Mr. Stuart de la Rue; Sir George Frampton, R.A.; Lord Glenconner; Viscount Goschen, C.B.E.; Mr. Charles Richard Hoare, Mr. H. B. Irving; Sir Woodburn Kirby; Mr. A. Heathcote Long; Mr. Julian G. Lousada; Mr. Basil Mayhew; Lt.-Col. H. E. du C. Norris; Mr. Cecil H. Oliverson; Mr. Adrian D. W. Pollock; Mr. George Roberts; Sir Samuel Scott, Bt., M.P.; Mr. W. Emuss Taylor; Lt.-Col. Sir Richard Temple, Bt.; Mr. Leslie R. Vigers; Brig.-Gen. The Hon. Charles Willoughby, C.B., C.M.G.; Mr. F. P. Whitbread; Mr. J. S. Wood; Mr. Ernest Woolley. Also Sir William Plender, G.B.E.; Mr. Edwin Freshfield, M.A.; the Rev. Prebendary Stone, M.A.; Mr. and Mrs. J. Arthur Rank, and Mr. H. R. Maynard (Secretary).

**A MEMORABLE OCCASION.**

In organising this dinner the Lord Mayor made a new departure. He dispensed with the attendance of the toast-master and decreed that there should be no speeches, two marked features of Mansion House dinners. This was a dinner of welcome to the Prince of Wales, as Heir-Apparent to the Throne, and the whole purpose was to make the occasion one calculated to give enjoyment to everybody present. Most of the guests were known to each other, many of them intimately, from having been associated together in the administration of the affairs of the Fund from the date of its foundation in 1897. The menu was simple but sufficient, and the wine of the best. The conversation during the dinner was well sustained, and although there were some hundred guests there were no dull or silent centres at the tables, but all went merrily as a marriage bell. The Lord Mayor drank the health of His Majesty the King, and proposed the health of the President of the Fund, His Royal Highness the Prince of Wales, in a few well-chosen and earnest words of welcome. His Royal Highness in an excellent little speech returned thanks, and proposed the health of the Lord Mayor and Lady Mayoress. In due course the Prince of Wales conducted the Lady Mayoress to the salon, where the principal guests were presented to His Royal Highness, and the party broke up into little groups. One and a-half hours were spent most helpfully, enabling the guests to mingle and discuss, whilst many who were seldom able to meet renewed old friendships and spent a most enjoyable, instructive, and delightful evening. Taken altogether it is not too much to say after fifty years of fairly active association with the City and Mansion House that the Banquet to His Royal Highness as President of the King's Hospital Fund will rank in importance, completeness, and success with the most successful and enjoyable assemblies ever brought together in the official residence of the Lord Mayor of London. The occasion was a memorable and attractively successful introduction to His Royal Highness the Prince of Wales, on succeeding to the Presidency of King Edward's Hospital Fund, an important office previous held by his Grandfather and his Father His Majesty King George V.

